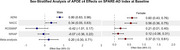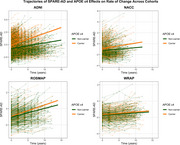# Sex‐Modified Effects of *APOE*‐ε4 on Spatial Patterns of Brain Atrophy: A Multi‐cohort Study

**DOI:** 10.1002/alz70856_103183

**Published:** 2025-12-26

**Authors:** Mengna Zhang, Guray Erus, Yuhan Cui, Shannon Risacher, Konstantinos Arfanakis, Duygu Tosun, Mohamad Habes, Di Wang, Arthur W. Toga, Paul M. Thompson, Walter W. Kukull, Sarah Biber, Bennett A. Landman, Barbara B. Bendlin, Sterling C. Johnson, Julie A Schneider, Lisa L. Barnes, David A. A. Bennett, Andrew J. Saykin, Michael L Cuccaro, Timothy J. Hohman, Christos Davatzikos, Derek Archer, Logan Dumitrescu, Alzheimer's Disease Neuroimaging Initiative (ADNI), The Alzheimer's Disease Sequencing Project (ADSP)

**Affiliations:** ^1^ Vanderbilt Memory and Alzheimer's Center, Vanderbilt University School of Medicine, Nashville, TN, USA; ^2^ University of Pennsylvania, Philadelphia, PA, USA; ^3^ Artificial Intelligence in Biomedical Imaging Laboratory, Perelman School of Medicine, University of Pennsylvania, Philadelphia, PA, USA; ^4^ Indiana Alzheimer's Disease Research Center, Indiana University School of Medicine, Indianapolis, IN, USA; ^5^ Department of Radiology and Imaging Sciences, Indiana University School of Medicine, Indianapolis, IN, USA; ^6^ Department of Biomedical Engineering, Illinois Institute of Technology, Chicago, IL, USA; ^7^ Rush Alzheimer's Disease Center, Rush University Medical Center, Chicago, IL, USA; ^8^ Department of Diagnostic Radiology and Nuclear Medicine, Rush UniversityRush University Medical Center, Chicago, IL, USA; ^9^ Department of Radiology and Biomedical Imaging, University of California, San Francisco, San Francisco, CA, USA; ^10^ Glenn Biggs Institute for Alzheimer's & Neurodegenerative Diseases, University of Texas Health Sciences Center at San Antonio, San Antonio, TX, USA; ^11^ UT Health Science Center at San Antonio, San Antonio, TX, USA; ^12^ Mark and Mary Stevens Neuroimaging and Informatics Institute, Keck School of Medicine, University of Southern California, Los Angeles, CA, USA; ^13^ Imaging Genetics Center, Mark and Mary Stevens Neuroimaging and Informatics Institute, Keck School of Medicine, University of Southern California, Marina del Rey, CA, USA; ^14^ National Alzheimer's Coordinating Center, University of Washington, Seattle, WA, USA; ^15^ Department of Neurology, Vanderbilt University Medical Center, Nashville, TN, USA; ^16^ Department of Computer Science, Vanderbilt University, Nashville, TN, USA; ^17^ Vanderbilt Brain Institute, Vanderbilt University Medical Center, Nashville, TN, USA; ^18^ Department of Electrical and Computer Engineering, Vanderbilt University, Nashville, TN, USA; ^19^ Department of Biomedical Engineering, Vanderbilt University, Nashville, TN, USA; ^20^ Department of Radiology and Radiological Sciences, Vanderbilt University Medical Center, Nashville, TN, USA; ^21^ Vanderbilt University Institute of Imaging Science, Vanderbilt University Medical Center, Nashville, TN, USA; ^22^ Wisconsin Alzheimer's Disease Research Center, School of Medicine and Public Health, University of Wisconsin, Madison, WI, USA; ^23^ Wisconsin Alzheimer's Disease Research Center, University of Wisconsin School of Medicine and Public Health, Madison, WI, USA; ^24^ Wisconsin's Alzheimer's Institute, School of Medicine and Public Health (SMPH), University of Wisconsin‐Madison, Madison, WI, USA; ^25^ Rush Alzheimer's Disease Center, Chicago, IL, USA; ^26^ Indiana Alzheimer's Disease Research Center, Indiana University School of Medicine, Indianapolis, IN, USA; ^27^ Department of Radiology and Imaging Sciences, Center for Neuroimaging, School of Medicine, Indiana University School of Medicine, Indianapolis, IN, USA; ^28^ Dr. John T. Macdonald Foundation Department of Human Genetics, University of Miami Miller School of Medicine, Miami, FL, USA; ^29^ The John P. Hussman Institute for Human Genomics, University of Miami, Miami, FL, USA; ^30^ Vanderbilt Memory and Alzheimer's Center, Vanderbilt University School of Medicine, Nashville, TN, USA; ^31^ Vanderbilt Genetics Institute, Vanderbilt University Medical Center, Nashville, TN, USA; ^32^ Department of Radiology, University of Pennsylvania, Philadelphia, PA, USA; ^33^ Department of Neurology, Vanderbilt Memory & Alzheimer's Center, Vanderbilt University Medical Center, Nashville, TN, USA

## Abstract

**Background:**

The SPARE‐AD (Spatial Pattern of Abnormalities for Recognition of Early Alzheimer's Disease [AD]) index effectively captures the level of AD‐association patterns of brain atrophy present in elderly individuals. *APOE* ε4, the strongest genetic risk factor for AD, demonstrates sex‐dependent effects with greater risk in females compared to males. Given SPARE‐AD's sensitivity to AD‐related brain changes, we investigated whether *APOE*‐ε4 carrier status influences brain atrophy patterns measured by SPARE‐AD and if these effects are modified by sex.

**Method:**

This study included 3,289 non‐Hispanic White participants (mean SPARE‐AD=‐0.79; mean age=72.3; 55.2% cognitive normal; 33.1% MCI; 11.1% AD; 54.1% female) from 4 AD and cognitive aging cohorts (ADNI, NACC, ROS/MAP, WRAP). The SPARE‐AD index was calculated using a high‐dimensional, non‐linear pattern classification method with positive values indicating AD‐like brain atrophy and negative values indicating normal brain structure. In cross‐sectional analyses at baseline, we evaluated the dominant effects of *APOE*‐ε4 carrier status on SPARE‐AD using multiple linear regression, adjusting for age, sex, and education levels. Sex‐stratified analyses were conducted to examine effect modification. In longitudinal analyses, we applied linear mixed‐effects models with time (years from baseline) and the intercept as fixed and random effects. We first evaluate the longitudinal progression of SPARE‐AD among cognitively unimpaired participants versus participants with MCI at baseline. We then assessed the effects of *APOE*‐ε4 carrier status on SPARE‐AD progression rates and their modification by sex. All analyses were meta‐analyzed across cohorts.

**Result:**

*APOE*‐ε4 carriers showed significantly higher SPARE‐AD indices at baseline versus non‐carriers (β=0.34; SE=0.16; *p* = 0.029), with this effect being significant only among females (β‐females=0.37; *p*‐females=0.0021; Figure 1). The utility of SPARE‐AD as a prodromal biomarker was validated in longitudinal analyses, where cognitively unimpaired individuals demonstrated significantly slower SPARE‐AD progression compared to those with MCI (β=‐0.11; SE=0.04; *p* = 0.0052). *APOE*‐ε4 carriers exhibited accelerated SPARE‐AD progression (β=0.05; SE=0.025; *p* = 0.047; Figure 2), with no sex differences observed.

**Conclusion:**

Our multi‐cohort study demonstrates that *APOE*‐ε4 carrier status influences both brain atrophy patterns and their progression. While baseline *APOE*‐ε4 effects were female‐specific, progression rates were sex‐independent. These findings advance our understanding of sex‐specific genetic influences on AD‐related brain changes.